# Investigating the effectiveness of water fluoridation

**DOI:** 10.1038/s41432-024-01032-4

**Published:** 2024-07-03

**Authors:** Darshini Ramasubbu, Jonathan Lewney, Brett Duane

**Affiliations:** 1 Dublin Dental University Hospital, Trinity College Dublin, Dublin, Republic of Ireland; 2 Independent Researcher, Paris, France

**Keywords:** Dental public health, Preventive dentistry

## Abstract

**Design:**

This retrospective cohort study used treatment claims data submitted over a 10-year period to explore the effect of water fluoridation on specified National Health Service (NHS) dental treatments, number of Decayed Missing and Filled Teeth (DMFT) and its cost-effectiveness. Ethical approval was granted and data was collected from NHS primary care settings via claims submitted to the NHS Business Services Authority (NHS BSA). To be included, participants must have attended dental services twice in the study period, been 12 years or over and had a valid English postcode. Those with claims related solely to orthodontic care were excluded, as were those who had requested NHS National Data Opt-out. Costs relating to water fluoridation were supplied by Public Health England. NHS BSA data was used to calculate NHS costs at 2020 prices.

**Cohort selection:**

A personalised water fluoride exposure for the 2010–2020 period was assigned to all individuals, who were then split into two groups, above 0.7 mg F/L (optimally fluoridated group) or lower (non-optimally fluoridated group). Individuals in each group were matched for analysis using propensity scores, estimated via logistic regression.

**Data analysis:**

Values of absolute standardised mean differences were used to determine covariate balance between the two groups, alongside a generalised linear model with matching weights and cluster robust standard errors and a patient deprivation decile as an interaction term. An Incremental Cost-Effectiveness Ratio (ICER) was calculated and differences in the overall costs to the public sector were illustrated by the return on investment estimate.

**Results:**

The cohort contained data on 6,370,280 individuals. Negative binomial regression models were used to analyse health outcomes. In the optimally fluoridated group, the rate of invasive dental treatments was 3% less than in the non-optimally fluoridated group, and the mean DMFT in the optimally fluoridated group was 2% lower. There was no evidence of a difference in the predicted mean number of missing teeth between groups. There was a small reduction in the predicted number of invasive treatments in the optimally fluoridated group but the largest predicted reduction was in the most deprived decile. DMFT did not exhibit the expected social inequalities gradient, and for the mean number of missing teeth there were small differences in each decile of deprivation between groups but the direct effect was inconsistent. Water fluoridation expenditure between 2010 and 2019 was estimated to be £10.30 for those receiving optimally fluoridated water. The marginal effects estimate illustrated savings of £22.26 per person (95% CI − £21.43, −£23.09), which is a relative reduction in costs to the NHS of 5.5% per patient. A subsequent estimation of cost effectiveness calculated the cost of water fluoridation to avoid one invasive dental treatment (the ICER) as £94.55. The estimated return on investment using a variety of NHS dental attendance estimates all lead to a positive return.

**Conclusions:**

These results suggest that water fluoridation appears to be producing less impactful effects on oral health, with water fluoridation resulting in ‘exceedingly small’ health effects and very small reductions in use of NHS dental services. A positive return for the public sector was identified as the costs of NHS dentistry are high and costs of water fluoridation are low, though this study did not include the original set up costs of fluoridation programmes.

## A Commentary on

**Moore D, Nyakutsikwa B, Allen T** **et al**.

How effective and cost-effective is water fluoridation for adults and adolescents? The LOTUS 10-year retrospective cohort study. *Community Dent Oral Epidemiol* 2024; 10.1111/cdoe.12930.

**GRADE Rating**: 

## Commentary

Community water fluoridation involves the addition of a fluoride compound to public water supplies, usually around 1ppm and is in place in approximately 25 countries^1^. It is considered a key strategy in the prevention of caries, benefiting whole populations, irrespective of socioeconomic status or dental attendance^1^.

The LOTUS study addressed a clearly focused issue; aiming to assess effectiveness of water fluoridation (intervention) in preventing dental treatment and improving oral health (outcomes) in adolescents 12 years or older and adults (population)^2^. This age group was specified as they were likely to have the majority of their adult teeth. The two groups for comparison were created depending on each individual’s personalised water fluoride exposure from 2010 to 2020^2^.

The ‘natural experiment’ was a retrospective cohort study. Retrospective cohort studies are advantageous in that they often use large sample sizes, include people who may be excluded from randomised controlled trials, and can be quick and inexpensive to undertake. This makes them useful in exploring population based interventions such as water fluoridation and their effects over a long time period^3^. Their limitations may include issues with a lack of randomisation, bias and confounding factors, which means their evidence is of a lower grade than that produced by randomised controlled trials^3^.

The cohort was recruited from claims submitted to the NHS Business Authority from 22nd November 2010 to 21st October 2020, and participants must have been 12 years or over at the start date^2^. As highlighted by the authors, this data was not initially gathered to assess the effectiveness of water fluoridation. However, using available data in this way is an innovative way to investigate a population based intervention^2^. It was highlighted that NHS numbers were traceable for approximately 70% of claims submitted and that data linkage issues more frequently affect those living in deprived areas, with no fixed abode and ethnic minorities^2^. The data extracted from records were not validated against an epidemiological record standard^2^. As the cohort was derived from those who had sought NHS dental treatment from a variety of primary care dental providers, it is representative of patients seeking and engaging with active treatment^2^.

The two groups of the cohort were split depending on if their personalised water fluoride exposure score was above 0.7 mg F/L (optimally fluoridated group) or lower (non- optimally fluoridated group)^2^. Considerable detail was supplied by the authors on how they calculated the exposure to fluoridated water of participants, the matching of the two groups and how confounding factors were accounted for^2^. The measurement of exposure to fluoridated water was calculated using individual’s Lower Super Output Area(s), which are census districts, and the annual mean drinking water fluoride concentrations (Mg F/L) for each year of the study^2^. Simple imputation was used if data was missing and the 0.7 mg F/L score was justified by previous research^2^. The patient’s personalised score was an estimate. Propensity scores, used to estimate the effect of water fluoridation by accounting for covariates that predict receiving water fluoridation, were estimated using logistic regression and used to create comparable groups for analysis^2,4^. This cohort contained a large sample of 6,370280 individuals and values of absolute standard mean difference were used to determine covariate balance^2^. There were more females, which may reflect higher use of dental services^2^.

The study investigated the effect of water fluoridation on clinical outcomes such as mean DMFT, missing teeth and number of invasive treatments, using a generalised linear model, which included deprivation deciles^2^. It concluded that the mean DMFT was 2% lower in the optimally fluoridated group, invasive treatments were less in the optimally fluoridated group, and that there was no difference in numbers of missing teeth between groups^2^. It was also noted that the DMFT did not exhibit the expected relationship with social gradient^2^. Data on water fluoridation expenditure was used to investigate cost savings and cost effectiveness, though it was not clear how patient payment contributions were factored in, as only saving in NHS treatment cost was reported^2^.

The overall conclusion suggests that the impact on oral health of water fluoridation seems to be reduced compared to previous, historical studies^2^. The results of the study highlight a need for more research in this area, particularly around some possible reasons for the observed effects and exploration of other methods to address the dental impacts of social inequalities.

The LOTUS study conclusions support the position that water fluoridation remains a cost saving public health intervention, which aligns with other research, including the recent CATFISH study^5^. The cost of setting up new schemes and their predicted impact needs further research and consideration, including from an environmental sustainability perspective^6^. It is also important to explore the relative effectiveness and cost-effectiveness of other methods of caries prevention such as reducing sugar consumption and health promotion practices and policies, particularly those that clearly reduce social inequalities.

Practice points

- Community water fluoridation was shown to be effective and cost-effective in reducing dental caries.
- Effect sizes are smaller than in previous, historical studies.
- A number of limitations exist and these were generally well-described by the authors.
